# Feeling Younger in Rural Burkina Faso: Exploring the Role of Subjective Age in the Light of Previous Research From High-Income Countries

**DOI:** 10.1093/geronb/gbab151

**Published:** 2021-08-11

**Authors:** Anton Schönstein, Dinh Thao Trinh Ngo, Yannick Stephan, Ali Siè, Guy Harling, Till Bärnighausen, Hans-Werner Wahl

**Affiliations:** 1 Network Aging Research, Heidelberg University, Heidelburg, Germany; 2 Heidelberg University, Heidelburg, Germany; 3 University of Montpellier, Montpelier, France; 4 CRSN Nouna, Boucle de Mouhoun, Burkina Faso; 5 University College of London, London, UK; 6 Network Aging Research and Institute of Psychology, Heidelberg University, Heidelberg, Germany

**Keywords:** Cross-cultural differences, Health, Stereotypes, Views on aging

## Abstract

**Objectives:**

Previous research on subjective age (SA), that is, how young or old a person feels relative to their chronological age, has shown that older adults tend to feel younger than they are (by about 15%–20%), but the extent of this effect depends, in part, on their health. However, as most of the studies have been conducted in Western countries, it is unclear how well these results generalize to culturally different samples. Objectives, therefore, were to examine SA in middle-aged and older adults from a very low-income setting in rural Burkina Faso, to examine associations between SA and health/quality of life-related measures, and to compare findings with Western studies.

**Methods:**

Representative, cross-sectional sample of *N* = 3,028 adults (≥40 years, recruited in 2018) from north-western Burkina Faso. Data included questionnaires on depression (Patient Health Questionnaire-9) and quality of life (World Health Organization Quality of Life scale, including subjective health) as well as performance-based and objective health-related measures (Community Screening Instrument for Dementia as cognitive screening, walking speed).

**Results:**

Respondents felt on average 3% younger (*SD* = 0.13) than their chronological age, with 48% (95% confidence interval: 0.46–0.50) feeling younger—27 percentage points lower than seen in representative Western studies. Lower depression, better walking speed, cognition, and quality of life were all associated with younger SA.

**Discussion:**

Middle-aged and older adults in Nouna felt less young than similar age groups in Western studies. One of the reasons may be that youthfulness is less of a value outside Western cultures. As in Western studies, parts of the variation in SA can be explained by health parameters.

Research on subjective age (SA) or felt age is driven by the fundamental assumption that how individuals interpret and evaluate their own aging is a major component of their identity and matters for their psychosocial adaptation (Diehl et al., 2014; Kastenbaum et al., 1972). Studies reported that older adults tend to feel younger than they are and that this discrepancy between felt age and chronological age increases as people grow older (Goldsmith & Heiens, 1992; Kaufman & Elder, 2002; Rubin & Berntsen, 2006). Rubin and Berntsen (2006) elaborate that, as in their sample young adolescents also reported to feel older, this pattern would be compatible with the idea of an *attractor age* toward which people of all ages strive when reporting how old they feel. According to them, this attractor age could be in early adulthood as this period is marked by a high density of life events (Berntsen & Rubin, 2004), autobiographical memories (Rubin et al. 1998), and carries special importance regarding a person’s identity (Conway & Pleydell-Pearce, 2000). Importantly Rubin and Berntsen (2006) concluded that the exact attractor age and maximum proportional discrepancy (about 20% in their sample) might differ in populations due to, for example, cultural factors, but the general pattern underlying the idea of an attractor age (increasing absolute discrepancy with distance from the attractor age) would hold true as a universal phenomenon.

It should be noted that already early work on the matter has considered SA to be multidimensional (Diehl et al., 2014; Kastenbaum et al., 1972). Kornadt et al. (2016) showed that SA cannot only differ across age groups but also across life domains (e.g., family, personality, finances, health). Additionally, emerging work beyond the construct of SA increasingly favors multidimensional approaches to assess self-perceptions of aging such as awareness of age-related gains versus losses (Diehl et al., 2021; Neupert & Bellingtier, 2017). Still, measuring SA as unidimensional has remained the common approach in the area and the bulk of available findings relies on this approach (Pinquart & Wahl, 2021).

Quantifying how many people in a population feel younger by how much has received considerable attention in the past. Kleinspehn-Ammerlahn et al. (2008) found that participants in the Berlin Aging Study felt on average 11% younger and the percentage further decreased to 8.5% across a 6-year longitudinal interval. In often-cited large-scale representative studies with comparable inclusion criteria (minimum age ≥40–50 years) conducted in Western countries (USA: Midlife in the United States, Health and Retirement Study; UK: English Longitudinal Study of Ageing), the proportion of participants reporting to feel younger was between 73% and 81% (Demakakos et al., 2007; Stephan et al., 2018; Westerhof & Barrett, 2005). In Rubin and Berntsen’s Danish sample, about 70% in that same age range reported feeling younger.

## SA in the Cross-Cultural and African Context

Barak (2009) compared SA across 18 countries and observed a general pattern of participants feeling younger. Most recently, the meta-analysis by Pinquart and Wahl (2021) based on 294 studies (with mean age ranging from 8 to 105 years) found a widening of the gap between SA and chronological age as people get older in all continents. Although adults reported a relatively younger SA across the globe, these differences were strongest in North America, Western Europe, and Australia/Oceania and weakest in Africa. Regional differences disappeared after statistically controlling for national levels of individualism–collectivism, power distance, preference for young people rather than older adults, and quality of life of older people.

Overall, as the recent meta-analysis by Pinquart and Wahl (2021) also supports, the bulk of previous research on SA builds on Western and to some extent Asian samples, whereas some research on African samples, our main target for this article, is available but in need of extension. Among the few studies in the African context is work by Macia et al. (2012) based on a representative sample of 500 dwellers of the Senegalese capital (aged ≥50 years). They found that 28% felt younger, 69% felt the same age, and 3% felt older. Still, 80% of the sample claimed to be “old.” Further research conducted by Macia et al. (2019) in Dakar, Senegal on a representative sample of 1,000 residents (≥20 years old) found that on average participants only felt about 1 year younger. Similarly, only 18% felt younger than they were; about 6% felt older; while the vast majority (76%) reported a felt age that matched their chronological age.

At the conceptual level, it has been argued that younger SA constitutes a compensatory strategy in cultures with predominantly negative age stereotypes (Schafer & Shippee, 2010). Cross-cultural researchers have referred to *modernization theory*, which states that modernization, often in the sense of industrialization, erodes the higher status associated with older age (Cowgill, 1974; Cowgill & Holmes, 1972). This may be especially relevant to countries like the ones that can be found in sub-Saharan Africa, where current socioeconomic transitions bring demographic aging and affect, among other areas, health care and financial security (National Academies of Sciences, Engineering, and Medicine, 2018; World Health Organization [WHO], 2020). A cross-cultural study that examined perceptions of aging across 26 cultures found that participants from Uganda, the only African country in the study, reported relatively positive societal views of aging; however, with the caveat that the proportion of older people in Uganda’s population structure was comparatively low (Löckenhoff et al., 2009). This might play a major role as North and Fiske (2015) found considerable support for the population aging–ageism hypothesis in their cross-cultural East–West comparison on attitudes toward older adults. Demographic aging processes and their consequences such as an overload of the health system could consequently increase negative attitudes toward aging, in turn facilitating the compensatory strategy to distance oneself from one’s SA in older age.

## SA and Health

An often-emphasized aspect about SA is that a considerable body of evidence has accumulated that points to the relevance of SA for health-related outcomes. Feeling younger was shown to be associated cross-sectionally, for example, with better functional health (Brothers et al., 2017) and lower disease burden (Choi et al., 2014; Spuling et al., 2013). In longitudinal studies, younger SA was associated with less overnight hospitalization (Stephan, Sutin, Terracciano, 2016), better sleep quality (Stephan et al., 2017), and reduced all-cause mortality (Levy et al., 2002; Stephan et al., 2018). Younger SA was also associated with lowered depression (Keyes & Westerhof, 2012; Spuling et al., 2013), higher subjective well-being (Steptoe et al., 2015; Westerhof & Barrett, 2005), and better objective and subjective memory functioning (Hülür et al., 2015; Stephan, Sutin, Caudroit et al., 2016).

Effects of SA on health may partly be explained by biological and behavioral pathways (Levy, 2009; Wurm et al., 2017). Feeling younger was linked to active health behavior (Wienert et al., 2015) and has also been found to be associated with reduced cystatin C indicating better kidney functioning (Stephan et al., 2019), as well as with benefits in terms of general biomarkers of aging (Wagner et al., 2016) such as C-reactive protein (Stephan et al., 2015b) and walking speed (Stephan et al., 2015a). The association of the more subjective health measures to SA may be stronger than to the more objective or performance-based measures (Schönstein et al., 2021).

Overall, a large body of evidence ties SA to health (Westerhof et al., 2014; Wurm et al., 2017) in participants from different countries and from midlife to advanced old age.

As with previous work, however, studies on the association between health and SA come to a considerable degree from Western countries and results from Africa have remained scarce. Nonetheless, Macia et al. (2012) could show that in their sample from Senegal, better subjective health was tied to younger SA. We will seek to extend these findings by employing a range of health measures in a poorer and more rural West African setting.

## Research Goals

The goals of this study are to provide a nuanced analysis of SA in a large sample of adults aged 40 years and older in rural Burkina Faso and to further provide a comparison with studies on SA conducted in Western countries. From a cross-cultural perspective, this means that we examine how SA acts in one of the poorest rural areas in the world.

First, following previous studies conducted in Africa (Macia et al., 2019) as well as meta-analytical work (Pinquart & Wahl, 2021), we hypothesize that the absolute discrepancy between felt age and chronological age, across the span of chronological age, will be less pronounced than in the often-cited study by Rubin and Berntsen (2006). Building on the idea of an *attractor age*, we likewise expect that this absolute discrepancy will be higher with increasing chronological age.

Second, and building on previous studies (Macia et al., 2012), we accordingly hypothesize that we will find a considerably smaller proportion of people reporting to feel younger when compared with similar large-scale Western studies with an effort to recruit representative populations and comparable inclusion criteria.

Third, and extending the previous work by Macia et al. (2012), we expect that a set of quality of life and health-related measures will be associated with SA (operationalized as proportional score). We will further explore the association between SA and specific diseases, for which information is available in the sample from rural Burkina Faso.

## Method

### Design and Sample

The study was conducted within the Centre for Research on Health in Nouna (CRSN)’s health and demographic surveillance system (HDSS) area containing 58 villages and the town of Nouna in north-western Burkina Faso. This district can be referred to as the Nouna area. The Nouna area is poor relative to the national average (Lietz et al., 2015). The CRSN Heidelberg Aging Study (CHAS) was conducted between May and July 2018 and aimed to assess the health status of older adults (≥40 years) in this setting, including cardiovascular risks, psychosocial constructs, and cognitive functioning (Odland et al., 2020; Witham et al., 2019). CHAS sampled 3,998 of approximately 18,000 age-eligible HDSS residents from the 2015 HDSS census in two parts. First, in the six villages with fewer than 50 eligible members, everyone was included. Second, everywhere else a random selection of households containing age-eligible individuals was made, and then one age-eligible person per selected household was included.

Potential participants were approached at their home for written informed consent (with a witness if illiterate). About 3,033 (76% of the originally sampled residents) agreed to participate. Participants then completed a structured questionnaire covering a range of topics, including physical and mental health, health care utilization, and social relations. Brief physical measurements were taken, and a venous blood draw was made. Data were collected using encrypted tablet computers. Clinical measures were assessed by trained and certified research staff. Interviews were conducted in the respondent’s preferred language, in 86% of cases, this was Dioula, with small minorities using French or one of the five other local languages.

Ethical approval for CHAS was obtained from the CRSN Comité d’éthique institutionnelle, the Comité national d’éthique pour la recherche en santé in Ouagadougou, and the Ethics Committee of the University of Heidelberg’s Faculty of Medicine.

### Measures: Subjective Age

SA was measured using a single item: “How old do you feel most of the time?” (Dutt et al., 2018; see Supplementary Material for French version). A proportional difference score $\;SA=\left(\frac{Felt\;\;\;Age\;-\;Chron.\;\;\;\;\;Age}{Chron.\;\;\;\;\;Age}\right)$, which informs about how much older or younger a person feels (in %), was calculated (Rubin & Berntsen, 2006). A negative score of, for example, −0.10 reflects someone feeling 10% younger than his or her chronological age. The usefulness of a single-item measure to assess SA is established in cross-cultural research (Barak, 2009).

The unrestricted SA format is prone to produce some unrealistic responses. Therefore, outliers were defined as observations with a proportional discrepancy of more than 3 *SD* from the variable’s mean (Stephan et al., 2018). These values (in total 65/3,028 or 2%) were set to “missing” to be handled by multiple imputation.

### Measures: Physical Health

*Walking speed* was measured as the amount of time a participant took to walk 4 m and back at their usual pace (Guralnik & Winograd, 1994). The test was conducted twice with the faster of the two walks used. Scores were adjusted for gender and height. Walking speed is a measure for functional mobility at large and predictive of future health outcomes (Guralnik & Winograd, 1994; LeBrasseur, 2019).

Information about several *specific diseases* (hypertension, diabetes, heart disease, chronic respiratory disease, tuberculosis, and stroke) was based on a self-report regarding objective information using questions of the format: “Has your health care worker ever informed you that you have heart disease?” Only diseases with *n* ≥ 30 cases in the sample were included. For hypertension and diabetes, respondents whose blood tests (capillary glucose >200 mg/dL, HbA1c >6.5%, or fasting glucose >126 mg/dL) or blood pressure examination (either systolic blood pressure ≥140 mmHg, or diastolic blood pressure ≥90 mmHg) indicated prevalent disease were also considered to have the condition.

### Measures: Mental Health

#### Affective health/depression

Depression was operationalized with the nine-item Patient Health Questionnaire (PHQ-9; Kroenke & Spitzer, 2002). Respondents rate the extent to which they have been bothered by symptoms indicating depression on a 4-point rating scale that reaches from 0 (*not at all*) to 3 (*nearly every day*). Data from East Africa (Gelaye et al., 2013) support its psychometric properties (Cronbach’s α = 0.81; retest reliability = 0.91). In this sample, Cronbach’s α was 0.80 (95% confidence interval [CI]: 0.79–0.81). The scale has shown its utility in assessing depression in older adults (Phelan et al., 2010).

#### Cognitive health

For assessment of cognitive health, the short version of the Community Screening Instrument for Dementia (CSI-D) was used, which was developed for cognitive screening purposes in cross-cultural research (Prince et al., 2011). Items include asking respondents to repeat three words after a defined interval (episodic memory) and asking them about their orientation. Previous research on similar items has shown their utility in African samples (Humphreys et al., 2017).

### Measures: Quality of Life

The WHO Quality of Life (WHOQOL)-Age Scale was used to measure quality of life (QoL). It has been found to offer a reliable and valid method to assess quality of life across a range of cultures (Caballero et al., 2013). The scale was shortened to eight items representing key domains for an African population in middle age and old age, that is, (a) quality of life at large, (b) satisfaction with health, (c) energy in everyday life, (d) satisfaction with the ability to perform activities of daily living, (e) satisfaction with oneself, (f) satisfaction with personal relationships, (g) sufficient money available, and (h) satisfaction with the living place. Answers were required on a 5-point Likert scale with higher values indicating better QoL. The scale achieved a Cronbach’s alpha of 0.91 in previous research (Caballero et al., 2013) and 0.80 (95% CI: 0.79–0.81) in this study.

### Statistical Analysis

For Hypothesis 1, we used a descriptive contrast of our results to the often-cited study of Rubin and Berntsen (2006). To mirror the approach by Rubin and Berntsen (2006), the Nouna sample was grouped into (chronological) age groups (each bin spanning 5 years), and these groups (*x*-axis) were then plotted against their associated felt age (*y*-axis) using means and standard deviations as summary measures.

To evaluate whether the proportion of people who feel younger is higher in Western than in African samples (Hypothesis 2), we displayed the respective proportions from comparable large-scale studies stratified by origin of the sample (African/Western) in a forest-plot figure and summarized the results with a random-intercept logistic regression model.

To examine the association of health variables with SA, we used hierarchical regression with a block-wise modeling approach similar to previous research (Schönstein et al., 2021) with SA (proportional score) as the outcome and the following predictors: (a) demographic variables (age, sex household size, and education); (b) functional mobility as measured by walking speed; (c) affective (PHQ-9) and cognitive (CSI-D) health; and (d) quality of life (WHOQOL). The respective age interactions of the health variables were also included accounting for a potential age dependency of effects.

We excluded those participants who lacked the basic assessment (i.e., missing value for gender), reducing the data set from 3,033 to a total of *N* = 3,028 observations. After this, the proportion of missing data across the data set was low (about 1% across all relevant cells, details given in Supplementary Table 1). For linear modeling, missing data were handled by multiple imputation (50 imputation data sets).

Statistical analyses were conducted using R version 3.6.1.

## Results

### Sample Description

Descriptive properties of the *N* = 3,028 sampled individuals who were located and consented to participate in the study are reported in Table 1. About 63% of participants described themselves as head of household. Regarding participants’ education, 84% answered that they had no formal schooling. About 8% reported less education than the primary school level while 5% reported completed education comparable to the level of primary school, about 1% completed education of the secondary school level. About 0.5% finished high school and 0.4% went to college or university. Zero-order correlations between the study’s main variables are presented in Supplementary Table 2.

**Table 1.** Descriptive Data of the Study Sample (*N* = 3,028)

Variables	*n* (%)	Mean (*SD*)	Median	Interquartile range (Q1, Q3)	Range (Min, Max)
*Demographic*					
Age		54.31 (11.0)	52	45, 62	40, 103
Sex male	1504 (50%)				
Sex female	1524 (50%)				
Household size		8.17 (5.4)	7	5, 10	1, 55
School years		1.01 (2.9)	0	0, 0	0, 38
*Physical health*					
Grip strength (kg)		37.95 (11.3)	37	30, 45	6, 79
BMI		22.06 (4.2)	21.41	19.26, 24.13	12.15, 45.83
Walking speed		0.97 (0.3)	1.00	0.80, 1.00	0.13, 2.00
Fried frailty score		0.86 (1.0)	1	0, 1	0, 5
WHODAS (norm.)		14.90 (17.4)	8.33	2.08, 22.92	0, 95.38
*Mental health*					
PHQ-9 score (depression)		4.38 (3.6)	4	2, 7	0, 23
CSI-D (cognition)		8.41 (1.1)	9	8, 9	2, 9
*Quality of life*					
WHOQOL (norm.)		56.01 (14.3)	59.38	46.88, 65.62	9.38, 96.88
*Subjective age measures*					
Felt age		52.81 (13.0)	50	43, 60	19, 100
Subjective age (diff.)		−1.50 (6.9)	0	−5, 1	−38, 28
Subjective age (prop.)		−0.03 (0.1)	0.00	−0.09, 0.02	−0.56, 0.46

*Notes:* Household size = total number of adults and children in the household; BMI = body mass index; WHODAS = WHO Disability Assessment Schedule with normalized scores (percentiles); PHQ-9 = Patient Health Questionnaire (nine-item version); CSI-D = Community Screening Instrument for Dementia; WHOQOL = WHO Quality of Life scale with normalized scores (percentiles); felt age = how old do you feel (years)?; Subjective age. (diff.) = Felt age − Chronological age; Subjective age (prop.) = (Felt age − Chronological age)/(Chronological age).

The average participant in the Nouna sample felt about one and a half years (95% CI: −1.75 to −1.25), or based on a proportional difference score of about 3% (95% CI: −0.033 to −0.024), younger. Around 30% of the sample felt older than they were, 21% felt the same age (in years) as their chronological age and 48% felt younger than they were.

### Hypothesis 1: Smaller Discrepancy Between SA and Chronological Age in the Nouna Sample Than in a Major Western Reference Study

**Figure 1.**
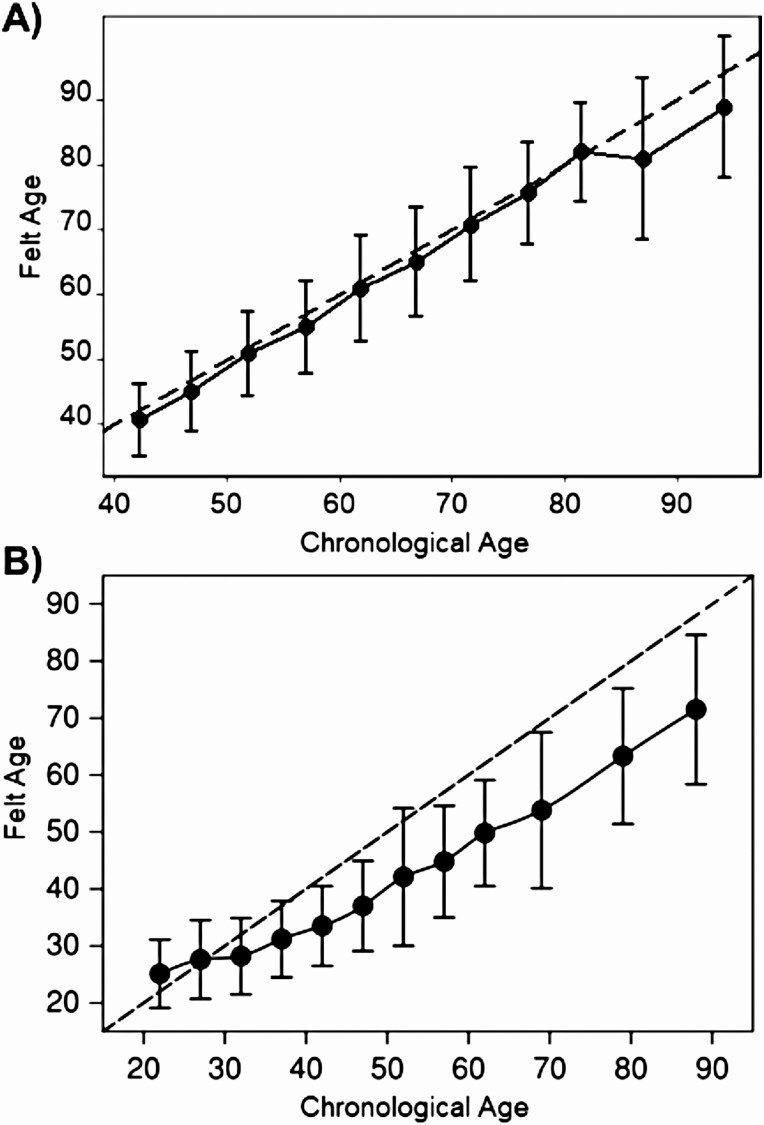
Felt age across the span of chronological age in (A) the Nouna Study (*N* = 3,028) and (B) in a Western reference by Rubin and Berntsen (*N* = 1,470; Rubin & Berntsen, 2006, p. 779). Dashed line functions as a reference that indicates Felt Age (in years; *y*-axis) equal to Chronological Age (in years; *x*-axis). In both (A) and (B) dots are means and whiskers show the standard deviation. Before the calculation of these descriptive summary measures, participants were grouped by chronological age (in bins of 5 years). Panel B is adapted from Rubin and Berntsen, 2006. Copyright [2006] by Springer Nature. Adapted with permission from Springer Nature Customer Center Service GmbH.

Figure 1 illustrates the small discrepancy between chronological age and felt age across chronological age in the Nouna sample when compared to the Western sample by Rubin and Berntsen (2006). The participants’ felt age barely deviated from their chronological age almost across all age groups. A larger deviation only occurs in “older old” females, that is, those aged older than 85, where the estimates are, however, less reliable due to the smaller number of cases (for sex-stratified analysis, see Supplementary Figure 1). Overall, the relationship between participants’ felt age and their chronological age appeared to be linear as can be taken from Supplementary Figure 2A, with the Pearson correlation amounting to *r* = 0.85 (95% CI: 0.84–0.86). In contrast, the discrepancy in the Rubin and Berntsen (2006) sample started to increase from age 40, and the gap surpassed 10 years by age 60.

### Hypothesis 2: Proportion of People Feeling Younger Is Smaller in the Nouna Sample Compared to Established Western Studies

**Figure 2.**
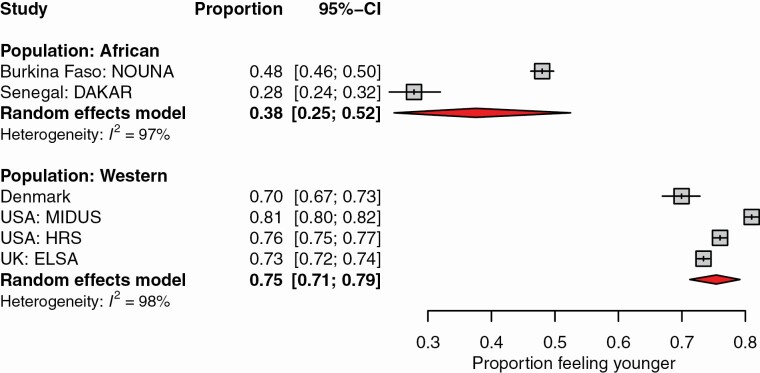
Proportion of participants feeling younger in the two African studies versus in a selection of comparable Western studies. Data are reported in Macia et al. (2012) for Senegal, Dakar; Rubin and Berntsen (2006) for the Danish sample; Stephan et al. (2018) for the USA: Midlife in the United States and Health and Retirement Study; and Demakakos et al. (2007) for the UK: English Longitudinal Study of Ageing. In all studies, the minimum recruitment age was 40–50 years, with an effort for a representative sample.

Following our reasoning in Hypothesis 1, we expected that the proportion of participants in the Nouna study who report feeling younger than they are would be smaller than in comparable representative samples of Western countries. For this purpose, we included established large studies from Western countries (Denmark, UK, and USA) with comparable inclusion criteria: recruitment of participants with a minimum age of 40–50 years, with the explicit effort for a representative sample (Demakakos et al., 2007; Stephan et al., 2018; Westerhof & Barrett, 2005). We also included the other comparable study from an African region that has been published, which recruited participants aged 50 years and older (Macia et al., 2012). A comparison of the proportions is displayed in Figure 2. Indeed, the proportion of individuals who felt younger was 37 percentage points higher in Western than in African samples. There was substantial heterogeneity among the African as well as Western studies.

### Hypothesis 3: Association of SA With Health-Related Indicators

#### Physical and mental health measures

Supplementary Figure 2B–F indicates that the relationship between SA (proportional score) and chronological age, walking speed, affective health, cognitive health, and quality of life, respectively, can reasonably be modeled as linear.

Table 2 presents the findings of hierarchical regression analyses for health-related measures predicting SA. Demographic variables (chronological age, sex, education, household size) explained only about 1% of variance in SA, with participants’ sex as the initially only statistically significant variable. Slower walking speed was associated with older SA, although again it explained only about 1% of additional variance. When affective (PHQ-9) and cognitive (CSI-D) health variables were added to this model, they added about 3% of explained variance. While both stronger symptoms of depression as well as symptoms of cognitive impairment were associated with older SA, affective health showed the strongest association to SA. Lower quality of life was associated with older SA and explained a further 1% of variance. None of the variables showed a statistically significant age interaction.

**Table 2.** Hierarchical Regression with Subjective Age (proportional score) as Outcome (*N* = 3,028)

	Model 1: Demographics		Model 2: Functional mobility		Model 3: Affective and cognitive health		Model 4: Quality of Life	
Predictor	β	95% CI: β	β	95% CI: β	β	95% CI: β	β	95% CI: β
Age	0.03	0.00, 0.07	0.00	−0.04, 0.04	−0.06**	−0.10, −0.02	−0.07**	−0.12, −0.03
Sex	0.08***	0.04, 0.11	0.04*	0.00, 0.08	0.02	−0.02, 0.05	0.01	−0.03, 0.05
Household size	−0.03	−0.07, 0.00	−0.02	−0.06, 0.01	−0.02	−0.05, 0.02	−0.01	−0.05, 0.03
Education	−0.02	−0.05, 0.02	−0.01	−0.05, 0.03	0.00	−0.04, 0.04	0.00	−0.03, 0.04
Walk speed			−0.12***	−0.16, −0.08	−0.10***	−0.14, −0.06	−0.09***	−0.13, −0.05
Walk speed × Age			0.01	−0.02, 0.05	0.02	−0.02, 0.05	0.02	−0.02, 0.05
PHQ-9					0.17***	0.13, 0.21	0.14***	0.10, 0.19
PHQ-9 × Age					0.00	−0.03, 0.03	0.01	−0.03, 0.05
CSI-D					−0.10***	−0.15, −0.06	−0.09***	−0.14, −0.05
CSI-D × Age					0.01	−0.02, 0.04	0.01	−0.03, 0.04
WHOQOL							−0.08***	−0.12, −0.04
WHOQOL × Age							0.02	−0.03, 0.06
*R*²	0.01		0.02		0.05		0.06	
Δ*R*^2^			0.01***		0.03***		0.01***	

*Notes:* PHQ-9 = Patient Health Questionnaire (nine-item version); CSI-D = Community Screening Instrument for Dementia; Household size = total number of adults and children in the household; WHOQOL = WHO Quality of Life scale with normalized scores (percentiles). β values are standardized regression coefficients. “Predictor × Age” denotes age interaction effects.

*p < .05, **p < .01, ***p < .001.

For illustrative purposes and to complement the data considering previous research by Macia et al. (2012), the negative association between the WHOQOL’s subjective health item and SA is also depicted in Supplementary Figure 3.

#### Associations between specific diseases and SA

**Figure 3.**
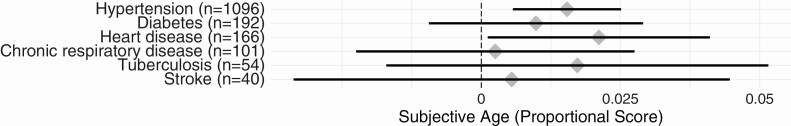
Estimates of the associations (with 95% CIs) between a range of specific diseases and Subjective Age as proportional score (*N* = 3,028). Estimates are controlled for demographics (age, sex, household size, education). For each estimate associated with a condition, the reference is participants without that condition. The number of cases for each disease in the data set is denoted by *n*. Only diseases with *n* ≥ 30 cases were included. Information on Hypertension and Diabetes was based on either objective physical examinations or self-report. Information on other diseases was based on self-report to a question of the format: Has your health care worker ever informed you that you have, e.g., heart disease?

After controlling for demographic variables, heart disease and hypertension were significantly associated with older SA (Figure 3): On average, participants with heart disease felt 1.5% older (95% CI: 0.01–0.03) and participants with hypertension felt about 2% older (95% CI: 0.00–0.04).

## Discussion

Previous research on SA has stated the need for more cross-cultural research (Barak, 2009; Macia et al., 2012; Montepare, 2009; Pinquart & Wahl, 2021). This article thus presents findings on SA based on a large representative sample of adults aged 40 years and older from Nouna, Burkina Faso.

First, we examined the discrepancy between felt age and chronological age across age groups. Participants from Nouna felt on average 1.5 years younger, which is considerably less than in Western samples. This result was closer to the 2.6-year younger discrepancy seen in a previous study conducted in Senegal (Macia et al., 2012) than the 6- to 12-year discrepancy seen in U.S. and European samples (Rubin & Berntsen, 2006; Stephan et al., 2018; Westerhof & Barrett, 2005). Expressed as a proportional difference score, Nouna participants felt about 3% younger than their age when compared with 14%–20% observed in several Western samples (Brothers et al., 2017; Rubin & Berntsen, 2006; Stephan et al., 2018).

One possible explanation for the variation in SA discrepancy across samples may be found in *modernization theory*. Senegal has a better human capital situation, taking rank 179 out of 195 countries in a study by Lim et al. (2018) compared to rank 193 of Burkina Faso. Denmark, the United States, and the United Kingdom rank 3rd, 27th, and 31st, respectively. As Löckenhoff et al. (2009) argue, especially education may play a vital role in socioeconomic growth often associated with less favorable perceptions of aging, which would thus be in accordance with the Senegalese sample reporting younger SA than participants from Burkina Faso. Beyond modernization theory, the considerable gap between Western and African samples in SA can also be seen as in accordance with the aging population–ageism hypothesis, as both Senegal and Burkina Faso have a demographic structure with considerably fewer older adults, although this may change in the future with the increasing life expectancy that we see in those countries.

We found a stronger linear relationship between the felt age (in years) and the chronological age in the Nouna sample (*r =* 0.85) than is typically seen in higher-income settings (e.g., *r* = 0.40 in Keyes & Westerhof, 2012). This is a stark contrast to the findings reported by Rubin and Berntsen (2006), where a stronger tendency toward younger SA with older chronological age was best summarized with a proportional score. While their results were compatible with the idea of a (culturally) variable *attractor age*, such a distinct pattern was not observable in our sample from Nouna, questioning the cross-cultural validity of this concept in its original form. Furthermore, our findings support data by Macia et al. (2019) who presented similar results, but without explicitly referring to the idea of an *attractor age*.

Our findings supported our hypothesis that studies from Africa would find a considerably smaller proportion of participants reporting younger SA than in Europe and the United States. These results can thus be seen in support of *modernization theory* (Cowgill, 1974; Cowgill & Holmes, 1972). It should, however, be noted that it appears inconsistent that the Dakar (Senegal) sample reported a lower rate of participants feeling younger than in the Nouna sample, given our argument about Senegal ranking better in the human capital situation (Lim et al., 2018). Notable heterogeneity in the African and Western studies should be considered, implying that this classification does not appear to be a sufficient explanation for the variation of study effects. This heterogeneity could be explained by more sophisticated meta-analytical approaches involving future work on SA in Africa as well as a variety of different cultures and a systematic review of the entire available literature. The recent meta-analysis by Pinquart and Wahl (2021) indicates that including information on national levels of orientation toward individualism/collectivism may help to explain some of the residual variations. With regard to the aging population–ageism hypothesis, information quantifying the extent of demographic aging in the respective nations should be involved in future meta-analytic approaches (North & Fiske, 2015).

Mostly in accordance with our Hypotheses 1 and 2, our findings support the notion that feeling younger than one’s chronological age (“youth orientation”) is an element more strongly represented in Western cultures when compared with African cultures. Still, the interpretation of our findings is far from unambiguous. It can be assumed that the reference of what a normal life span means may lead to a different mental scaling and awareness of aging likely starts much earlier in Burkina Faso. In fact, Bezzina (2019) reported that in Burkina Faso one is regarded as “old” from about 40 years onward. We may thus have compared an “old” sample from Nouna with middle adulthood *and* old age from the Western hemisphere. Future research might examine the issue of culturally different mental scaling, for example, by involving measures on subjective time-to-death alongside SA and by comparing these across different cultures. As opposed to approaches based on chronological age, switching to a time-to-death perspective has provided a more suitable explanation in previous research focusing on late-life development (Kotter-Grühn et al., 2009; Vogel et al., 2013).

Another issue of cross-cultural importance is that older adults in the Western hemisphere may be motivated to exert an “age bias,” assuming that feeling younger is a form of self-enhancement (Teuscher, 2009). However, old age may not be seen as negatively in Burkina Faso as it is in Western countries (Ng et al., 2015). Older adults in the Nouna sample were typically seen as the head of their household (de Jong, 2009). Given the knowledge on aging and what it means in Burkina Faso, differences found in SA may indeed indicate more positive aging views. Pinpointing the exact reason(s) for why aging views appear to be more positive in Burkina Faso is, however, beyond the scope of this article: A simply more accepting culture toward older people, a comparably less progressed industrialization which has therefore not undermined older people’s status, or comparably less population aging which has therefore not put older people as much into the focus of societal struggles, all present viable options (see also North & Fiske, 2015).

Finally, our findings also support that health and quality of life variables can, to a certain degree, explain the discrepancy between SA and chronological age (Hypothesis 3). Functional mobility, affective and cognitive health, and quality of life were all associated with SA when controlling for demographic variables. To date, numerous Western studies on aging views support Levy’s (2009) approach to stereotype embodiment theory, according to which physiological processes, behavioral pathways, and psychological mechanisms connect aging views to health (see also Wurm et al., 2017). Our findings from rural Burkina Faso extend the evidence for associations between health and SA to a culturally different context. Depressive symptoms showed the relatively strongest association to SA in our sample, complementing numerous findings that point to such an interrelation not only across longer time intervals but also on a day-to-day level (Bergland et al., 2014; Bodner et al., 2020; Keyes & Westerhof, 2012). It especially complements recent work, in which depression likewise appeared to be the relatively strongest factor (Hwang & Hong, 2019; Schönstein et al., 2021). This may point to a stronger role of subjective as opposed to objective or performance-based measures when explaining SA (Schönstein et al., 2021), although quality of life and walking speed, by definition examples for each a very subjective and a very objective measure, showed similarly strong associations with SA and thus results are not entirely consistent. In this context, it is worth pointing out that the public health situation continues to be problematic in Burkina Faso, and the view that external evil power may cause disease is common, particularly in rural regions (Bezzina, 2019). Therefore, morbidity may generally not be seen as a mostly age-related occurrence and subjective interpretation of disease, in general, might differ when compared to Western countries. It is also worth pointing out that the overall amount of explained variance was low: The full model involving demographic characteristics, functional mobility, affective and cognitive health, as well as quality of life, only explained about 6% of variance in SA. This raises the question of other factors that could explain SA variation. Indeed, health status provides at best very limited information on personal and developmental reference points that are, beyond the concept of an attractor age, supposedly involved in anchoring one’s SA (Diehl et al., 2014). Future research could address this by involving data on interpersonal social aspects and/or major life events, such as the death of a family member.

In terms of morbidity, hypertension and heart disease were also associated with older SA, which in light of research in terms of the burden of the disease in African countries (Soubeiga et al., 2017; WHO, 2004) and already shown associations in Western samples is in agreement with the previous literature (Diehr et al., 2001; Wurm et al., 2020).

### Limitations

The cross-sectional design does not allow for any causal inferences or even definite conclusions regarding SA’s measurement invariance across cultures, that is, whether feeling like a certain age is interpreted similarly by the participants.

Although French is the official language in Burkina Faso at least five other languages are commonly spoken in the study area and oral translation of questionnaires into these languages by interviewers may have caused variation in comprehension. Also, the participants might have never previously thought about their SA. The concept of SA is more common in the Western hemisphere as qualitative research indicates (Petery, 2018), and as such a differentiation is not as widespread in Burkina Faso it may explain why many participants simply respond to identify with their chronological age or anchor their SA closer to it. Although several cross-cultural studies have been conducted on SA and related constructs (Barak, 2009; Löckenhoff et al., 2009; Macia et al., 2012), the cross-cultural validity issue of measuring SA remains a challenge. Considering that there is evidence that supports the multidimensionality of SA (Kornadt et al., 2016), the use of a single-item measure should be seen as a limitation of this study. Future research could address this by incorporating multidimensionality in the measurement of SA. Culture-specific weighting in the importance of subdomains in SA may in the future help to explain discrepancies in SA between countries. Information on multidimensional SA would further also provide a more differentiated background on how different cultures qualitatively define SA and thus also allow for some conclusions in terms of cross-cultural measurement invariance of the construct.

In terms of measurement invariance at the predictor level, we strived to consider only instruments that have shown cross-cultural validity. SA has already seen a range of cross-cultural studies (Macia et al., 2012) and the similarities in associations between SA and health may also be regarded as partial support for the assumption of measurement invariance.

## Conclusions

By studying SA in a rural Burkina Faso setting, we raise several important issues, some of which have, to the best of our knowledge, been explored in an African sample for the first time. First, the pattern of differences between felt age and chronological age that we found calls into question the concept of an *attractor age*, which was originally derived from Western samples. Second, the infrequency with which Burkinabe respondents identified with a younger age may indicate variation in positive aging views across contexts. Third, this study provides evidence of intercultural validity for the association between SA and health, especially for the association between depression and SA. Future research should employ longitudinal designs to be able to address the challenge of SA’s measurement invariance across cultures and to solidify the directionality of effects found in this study.

## Supplementary Material

gbab151_suppl_Supplementary_Materials_1

## References

[CIT0001] Barak, B . (2009). Age identity: A cross-cultural global approach. International Journal of Behavioral Development, 33(1), 2–11. doi:10.1177/0165025408099485

[CIT0002] Bergland, A., Nicolaisen, M., & Thorsen, K. (2014). Predictors of subjective age in people aged 40–79 years: A five-year follow-up study. The impact of mastery, mental and physical health. Aging & Mental Health, 18(5), 653–661. doi:10.1080/13607863.2013.869545 24359016

[CIT0003] Berntsen, D., & Rubin, D. C. (2004). Cultural life scripts structure recall from autobiographical memory. Memory & Cognition, 32(3), 427–442. doi:10.3758/BF03195836 15285126

[CIT0004] Bezzina, L . (2019). Disability and development in Burkina Faso: Critical perspectives. Springer Nature.

[CIT0005] Bodner, E., Shrira, A., Hoffman, Y., & Bergman, Y. S. (2020). Day-to-day variability in subjective age and ageist attitudes and their association with depressive symptoms. The Journals of Gerontology, Series B: Psychological Sciences and Social Sciences, 76(5), 836–844. doi:10.1093/geronb/gbaa125 32808666

[CIT0006] Brothers, A., Miche, M., Wahl, H.-W., & Diehl, M. (2017). Examination of associations among three distinct subjective aging constructs and their relevance for predicting developmental correlates. The Journals of Gerontology, Series B: Psychological Sciences and Social Sciences, 72(4), 547–560. doi:10.1093/geronb/gbv085 PMC5927160 26430165

[CIT0007] Caballero, F. F., Miret, M., Power, M., Chatterji, S., Tobiasz-Adamczyk, B., Koskinen, S., Leonardi, M., Olaya, B., Haro, J. M., & Ayuso-Mateos, J. L. (2013). Validation of an instrument to evaluate quality of life in the aging population: WHOQOL-AGE. Health and Quality of Life Outcomes, 11, 177. doi:10.1186/1477-7525-11-177 24152691 PMC4015924

[CIT0008] Choi, N. G., DiNitto, D. M., & Kim, J. (2014). Discrepancy between chronological age and felt age: Age group difference in objective and subjective health as correlates. Journal of Aging and Health, 26(3), 458–473. doi:10.1177/0898264314523449 24583944 PMC4564247

[CIT0009] Conway, M. A., & Pleydell-Pearce, C. W. (2000). The construction of autobiographical memories in the self-memory system. Psychological Review, 107(2), 261–288. doi:10.1037/0033-295x.107.2.261 10789197

[CIT0010] Cowgill, D. O . (1974). The aging of populations and societies. The Annals of the American Academy of Political and Social Science, 415(1), 1–18. doi:10.1177/000271627441500102

[CIT0011] Cowgill, D. O., & Holmes, L. D. (1972). Aging and modernization. Appleton-Century-Crofts and Fleschner Publishing Company.

[CIT0012] de Jong, W . (2009). Altern in Unsicherheit. In Kerala wie in Burkina Faso erweist sich die Alterssicherung über die Grossfamilie als Mythos. Welt-Sichten, 4, 12–16. doi:10.5167/uzh-30982

[CIT0013] Demakakos, P., Gjonca, E., & Nazroo, J. (2007). Age identity, age perceptions, and health: Evidence from the English Longitudinal Study of Ageing. Annals of the New York Academy of Sciences, 1114, 279–287. doi:10.1196/annals.1396.021 17986588

[CIT0014] Diehl, M., Brothers, A. F., & Wahl, H.-W.(2021). Self-perceptions and awareness of aging: Past, present, and future. In K. W. Schaie & S. L. Willis (Eds.), Handbook of psychology of aging (9th ed.). Academic Press.

[CIT0015] Diehl, M., Wahl, H. W., Barrett, A. E., Brothers, A. F., Miche, M., Montepare, J. M., Westerhof, G. J., & Wurm, S. (2014). Awareness of aging: Theoretical considerations on an emerging concept. Developmental Review, 34(2), 93–113. doi:10.1016/j.dr.2014.01.001 24958998 PMC4064469

[CIT0016] Diehr, P., Williamson, J., Patrick, D. L., Bild, D. E., & Burke, G. L. (2001). Patterns of self-rated health in older adults before and after sentinel health events. Journal of the American Geriatrics Society, 49(1), 36–44. doi:10.1046/j.1532-5415.2001.49007.x 11207840

[CIT0017] Dutt, A. J., Wahl, H.-W., & Diehl, M. (2019). Awareness of aging processes and the aging self. In O. Braddick (Ed.), Oxford Research Encyclopedia of Psychology (pp. 1053–1072). New York, NY: Oxford University Press. doi:10.1093/acrefore/9780190236557.013.397

[CIT0018] Gelaye, B., Williams, M. A., Lemma, S., Deyessa, N., Bahretibeb, Y., Shibre, T., Wondimagegn, D., Lemenhe, A., Fann, J. R., Vander Stoep, A., & Andrew Zhou, X. H. (2013). Validity of the Patient Health Questionnaire-9 for depression screening and diagnosis in East Africa. Psychiatry Research, 210(2), 653–661. doi:10.1016/j.psychres.2013.07.015 23972787 PMC3818385

[CIT0019] Goldsmith, R. E., & Heiens, R. A. (1992). Subjective age: A test of five hypotheses. The Gerontologist, 32(3), 312–317. doi:10.1093/geront/32.3.312 1499995

[CIT0020] Guralnik, J. M., & Winograd, C. H. (1994). Physical performance measures in the assessment of older persons. Aging (Milan, Italy), 6(5), 303–305. doi:10.1007/BF03324256 7893776

[CIT0021] Hülür, G., Hertzog, C., Pearman, A. M., & Gerstorf, D. (2015). Correlates and moderators of change in subjective memory and memory performance: Findings from the Health and Retirement Study. Gerontology, 61(3), 232–240. doi:10.1159/000369010 25790970

[CIT0071] Humphreys, G. W., Duta, M. D., Montana, L., Demeyere, N., McCrory, C., Rohr, J., Kahn, K., Tollman, S., & Berkman, L. (2017). Cognitive function in low-income and low-literacy settings: validation of the tablet-based Oxford cognitive screen in the health and aging in Africa: a longitudinal study of an INDEPTH community in South Africa (HAALSI). The Journals of Gerontology. Series B, Psychological Sciences and Social Sciences, 72(1), 38–50. doi:10.1093/geronb/gbw139 PMC5156498 27974474

[CIT0022] Hwang, Y., & Hong, G. S. (2019). Predictors of subjective age in community-dwelling older adults in Korea. Geriatric Nursing (New York, N.Y.), 40(3), 314–319. doi:10.1016/j.gerinurse.2018.11.008 30554730

[CIT0023] Kastenbaum, R., Derbin, V., Sabatini, P., & Artt, S. (1972). “The ages of me”: Toward personal and interpersonal definitions of functional aging. Aging and Human Development, 3(2), 197–211. doi:10.2190/TUJR-WTXK-866Q-8QU7

[CIT0024] Kaufman, G., & Elder, G. H. Jr (2002). Revisiting age identity: A research note. Journal of Aging Studies, 16(2), 169–176. doi:10.1016/S0890-4065(02)00042-7

[CIT0025] Keyes, C. L., & Westerhof, G. J. (2012). Chronological and subjective age differences in flourishing mental health and major depressive episode. Aging & Mental Health, 16(1), 67–74. doi:10.1080/13607863.2011.596811 21780972

[CIT0026] Kleinspehn-Ammerlahn, A., Kotter-Grühn, D., & Smith, J. (2008). Self-perceptions of aging: Do subjective age and satisfaction with aging change during old age? The Journals of Gerontology, Series B: Psychological Sciences and Social Sciences, 63(6), 377–385. doi:10.1093/geronb/63.6.p377 19092041

[CIT0027] Kornadt, A. E., Hess, T. M., Voss, P., & Rothermund, K. (2016). Subjective age across the life span: A differentiated, longitudinal approach. The Journals of Gerontology, Series B: Psychological Sciences and Social Sciences, 73(5), 767–777. doi:10.1093/geronb/gbw072 27334638

[CIT0028] Kotter-Grühn, D., Kleinspehn-Ammerlahn, A., Gerstorf, D., & Smith, J. (2009). Self-perceptions of aging predict mortality and change with approaching death: 16-year longitudinal results from the Berlin aging study. Psychology and Aging, 24(3), 654–667. doi:10.1037/a0016510 19739922

[CIT0072] Kroenke, K., & Spitzer, R. L. (2002). The PHQ-9: A new depression diagnostic and severity measure. Psychiatric Annals, 32(9), 509–515. doi:10.3928/0048-5713-20020901-06

[CIT0029] LeBrasseur, N. K . (2019). Gait as an integrative measure and predictor of health across species. The Journals of Gerontology, Series A: Biological Sciences and Medical Sciences, 74(9), 1411–1412. doi:10.1093/gerona/glz121 31074770

[CIT0030] Levy, B . (2009). Stereotype embodiment: A psychosocial approach to aging. Current Directions in Psychological Science, 18(6), 332–336. doi:10.1111/j.1467-8721.2009.01662.x 20802838 PMC2927354

[CIT0031] Levy, B. R., Slade, M. D., Kunkel, S. R., & Kasl, S. V. (2002). Longevity increased by positive self-perceptions of aging. Journal of Personality and Social Psychology, 83(2), 261–270. doi:10.1037//0022-3514.83.2.261 12150226

[CIT0032] Lietz, H., Lingani, M., Sié, A., Sauerborn, R., Souares, A., & Tozan, Y. (2015). Measuring population health: Costs of alternative survey approaches in the Nouna Health and Demographic Surveillance System in rural Burkina Faso. Global Health Action, 8, 28330. doi:10.3402/gha.v8.28330 26257048 PMC4530139

[CIT0033] Lim, S. S., Updike, R. L., Kaldjian, A. S., Barber, R. M., Cowling, K., York, H., Friedman, J., Xu, R., Whisnant, J. L., Taylor, H. J., Leever, A. T., Roman, Y., Bryant, M. F., Dieleman, J., Gakidou, E., & Murray, C. J. L. (2018). Measuring human capital: A systematic analysis of 195 countries and territories, 1990–2016. Lancet (London, England), 392(10154), 1217–1234. doi:10.1016/S0140-6736(18)31941-X PMC7845481 30266414

[CIT0034] Löckenhoff, C. E., De Fruyt, F., Terracciano, A., McCrae, R. R., De Bolle, M., Costa, P. T. Jr, Aguilar-Vafaie, M. E., Ahn, C. K., Ahn, H. N., Alcalay, L., Allik, J., Avdeyeva, T. V., Barbaranelli, C., Benet-Martinez, V., Blatný, M., Bratko, D., Cain, T. R., Crawford, J. T., Lima, M. P., … Yik, M. (2009). Perceptions of aging across 26 cultures and their culture-level associates. Psychology and Aging, 24(4), 941–954. doi:10.1037/a0016901 20025408 PMC2933107

[CIT0035] Macia, E., Dial, F. B., Montepare, J. M., Hane, F., & Duboz, P. (2019). Ageing and the body: One African perspective. Ageing & Society, 39(4), 815–835. doi:10.1017/S0144686X17001313

[CIT0036] Macia, E., Duboz, P., Montepare, J. M., & Gueye, L. (2012). Age identity, self-rated health, and life satisfaction among older adults in Dakar, Senegal. European Journal of Ageing, 9(3), 243–253. doi:10.1007/s10433-012-0227-7 28804424 PMC5547411

[CIT0074] Montepare, J. M . (2009). Subjective age: Toward a guiding lifespan framework. International Journal of Behavioral Development, 33(1), 42–46. doi:10.1177/0165025408095551

[CIT0037] National Academies of Sciences, Engineering, and Medicine . (2018). Future directions for the demography of aging: Proceedings of a workshop, Washington, DC: The National Academies Press. doi:10.17226/25064 29989766

[CIT0038] Neupert, S. D., & Bellingtier, J. A. (2017). Aging attitudes and daily awareness of age-related change interact to predict negative affect. The Gerontologist, 57(Suppl. 2), S187–S192. doi:10.1093/geront/gnx055 28854606

[CIT0039] Ng, R., Allore, H. G., Trentalange, M., Monin, J. K., & Levy, B. R. (2015). Increasing negativity of age stereotypes across 200 years: Evidence from a database of 400 million words. PLoS One, 10(2), e0117086. doi:10.1371/journal.pone.0117086 25675438 PMC4326131

[CIT0040] North, M. S., & Fiske, S. T. (2015). Modern attitudes toward older adults in the aging world: A cross-cultural meta-analysis. Psychological Bulletin, 141(5), 993–1021. doi:10.1037/a0039469 26191955

[CIT0041] Odland, M. L., Payne, C., Witham, M. D., Siedner, M. J., Bärnighausen, T., Bountogo, M., Coulibaly, B., Geldsetzer, P., Harling, G., Manne-Goehler, J., Ouermi, L., Sie, A., & Davies, J. I. (2020). Epidemiology of multimorbidity in conditions of extreme poverty: A population-based study of older adults in rural Burkina Faso. BMJ Global Health, 5(3), e002096. doi:10.1136/bmjgh-2019-002096 PMC7170422 32337079

[CIT0042] Petery, G. A . (2018). Developing a broader understanding of subjective age: A mixed methods investigation. https://opencommons.uconn.edu/dissertations/1727

[CIT0043] Phelan, E., Williams, B., Meeker, K., Bonn, K., Frederick, J., Logerfo, J., & Snowden, M. (2010). A study of the diagnostic accuracy of the PHQ-9 in primary care elderly. BMC Family Practice, 11, 63. doi:10.1186/1471-2296-11-63 20807445 PMC2940814

[CIT0044] Pinquart, M., & Wahl, H. W. (2021). Subjective age from childhood to advanced old age: A meta-analysis. Psychology and Aging, 36(3), 394–406. doi:10.1037/pag0000600 33829847

[CIT0045] Prince, M., Acosta, D., Ferri, C. P., Guerra, M., Huang, Y., Jacob, K. S., Llibre Rodriguez, J. J., Salas, A., Sosa, A. L., Williams, J. D., & Hall, K. S.; 10/66 Dementia Group. (2011). A brief dementia screener suitable for use by non-specialists in resource poor settings—The cross-cultural derivation and validation of the brief Community Screening Instrument for Dementia. International Journal of Geriatric Psychiatry, 26(9), 899–907. doi:10.1002/gps.2622 21845592 PMC3427892

[CIT0073] Rubin, D. C., Rahhal, T. A., & Poon, L. W. (1998). Things learned in early adulthood are remembered best. Memory & Cognition, 26(1), 3– 19. doi:10.3758/BF03211366 9519693

[CIT0046] Rubin, D. C., & Berntsen, D. (2006). People over forty feel 20% younger than their age: Subjective age across the lifespan. Psychonomic Bulletin & Review, 13(5), 776–780. doi:10.3758/bf03193996 17328372 PMC3969748

[CIT0047] Schafer, M. H., & Shippee, T. P. (2010). Age identity, gender, and perceptions of decline: Does feeling older lead to pessimistic dispositions about cognitive aging? The Journals of Gerontology, Series B: Psychological Sciences and Social Sciences, 65(1), 91–96. doi:10.1093/geronb/gbp046 19515992

[CIT0048] Schönstein, A., Dallmeier, D., Denkinger, M., Rothenbacher, D., Klenk, J., Bahrmann, A., & Wahl, H.-W. (2021). Health and subjective views on aging: Longitudinal findings from the ActiFE Ulm Study. The Journals of Gerontology, Series B: Psychological Sciences and Social Sciences, 76(7), 1349–1359. doi:10.1093/geronb/gbab023 PMC8363042 33528511

[CIT0049] Soubeiga, J. K., Millogo, T., Bicaba, B. W., Doulougou, B., & Kouanda, S. (2017). Prevalence and factors associated with hypertension in Burkina Faso: A countrywide cross-sectional study. BMC Public Health, 17(1), 64. doi:10.1186/s12889-016-3926-8 28077112 PMC5225558

[CIT0050] Spuling, S. M., Miche, M., Wurm, S., & Wahl, H.-W. (2013). Exploring the causal interplay of subjective age and health dimensions in the second half of life. Zeitschrift für Gesundheitspsychologie, 21(1), 5–15. doi:10.1026/0943-8149/a000084

[CIT0051] Stephan, Y., Sutin, A. R., Bayard, S., & Terracciano, A. (2017). Subjective age and sleep in middle-aged and older adults. Psychology & Health, 32(9), 1140–1151. doi:10.1080/08870446.2017.1324971 28480746

[CIT0052] Stephan, Y., Sutin, A. R., Caudroit, J., & Terracciano, A. (2016). Subjective age and changes in memory in older adults. The Journals of Gerontology, Series B: Psychological Sciences and Social Sciences, 71(4), 675–683. doi:10.1093/geronb/gbv010 25748213

[CIT0053] Stephan, Y., Sutin, A. R., & Terracciano, A. (2015a). “Feeling younger, walking faster”: Subjective age and walking speed in older adults. Age (Dordrecht, Netherlands), 37(5), 86. doi:10.1007/s11357-015-9830-9 PMC5005834 26296609

[CIT0054] Stephan, Y., Sutin, A. R., & Terracciano, A. (2015b). Younger subjective age is associated with lower C-reactive protein among older adults. Brain, Behavior, and Immunity, 43, 33–36. doi:10.1016/j.bbi.2014.07.019 25108213

[CIT0055] Stephan, Y., Sutin, A. R., & Terracciano, A. (2016). Feeling older and risk of hospitalization: Evidence from three longitudinal cohorts. Health Psychology, 35(6), 634–637. doi:10.1037/hea000033526867044

[CIT0056] Stephan, Y., Sutin, A. R., & Terracciano, A. (2018). Subjective age and mortality in three longitudinal samples. Psychosomatic Medicine, 80(7), 659–664. doi:10.1097/PSY.0000000000000613 29864106 PMC6345273

[CIT0057] Stephan, Y., Sutin, A. R., & Terracciano, A. (2019). Subjective age and cystatin C among older adults. The Journals of Gerontology, Series B: Psychological Sciences and Social Sciences, 74(3), 382–388. doi:10.1093/geronb/gbx124 PMC6377033 29045722

[CIT0058] Steptoe, A., Deaton, A., & Stone, A. A. (2015). Subjective wellbeing, health, and ageing. Lancet (London, England), 385(9968), 640–648. doi:10.1016/S0140-6736(13)61489-0 PMC4339610 25468152

[CIT0059] Teuscher, U . (2009). Subjective age bias: A motivational and information processing approach. International Journal of Behavioral Development, 33(1), 22–31. doi:10.1177/0165025408099487

[CIT0060] Vogel, N., Schilling, O. K., Wahl, H. W., Beekman, A. T., & Penninx, B. W. (2013). Time-to-death-related change in positive and negative affect among older adults approaching the end of life. Psychology and Aging, 28(1), 128–141. doi:10.1037/a0030471 23106152

[CIT0061] Wagner, K.-H., Cameron-Smith, D., Wessner, B., & Franzke, B. (2016). Biomarkers of aging: From function to molecular biology. Nutrients, 8(6), 338. doi:10.3390/nu8060338 PMC4924179 27271660

[CIT0062] Westerhof, G. J., & Barrett, A. E. (2005). Age identity and subjective well-being: A comparison of the United States and Germany. The Journals of Gerontology, Series B: Psychological Sciences and Social Sciences, 60(3), 129–136. doi:10.1093/geronb/60.3.s129 15860789

[CIT0063] Westerhof, G. J., Miche, M., Brothers, A. F., Barrett, A. E., Diehl, M., Montepare, J. M., Wahl, H. W., & Wurm, S. (2014). The influence of subjective aging on health and longevity: A meta-analysis of longitudinal data. Psychology and Aging, 29(4), 793–802. doi:10.1037/a0038016 25365689

[CIT0064] WHO . (2004). The atlas of heart disease and stroke, Geneva: World Health Organization.

[CIT0065] WHO . (2020). *The work of WHO in the African Region—Report of the Regional Director*. https://www.afro.who.int/publications/work-world-health-organization-african-region-report-regional-director-1-july-2019-30

[CIT0066] Wienert, J., Kuhlmann, T., & Lippke, S. (2015). Direct effects of a domain-specific subjective age measure on self-reported physical activity—Is it more important how old you are or how old you feel? Health Psychology Report, 3(2), 131–139. doi:10.5114/hpr.2015.51450

[CIT0067] Witham, M. D., Davies, J. I., Bärnighausen, T., Bountogo, M., Manne-Goehler, J., Payne, C. F., Ouermi, L., Sie, A., Siedner, M. J., & Harling, G. (2019). Frailty and physical performance in the context of extreme poverty: A population-based study of older adults in rural Burkina Faso. Wellcome Open Research, 4, 135. doi:10.12688/wellcomeopenres.15455.1 32280791 PMC7137808

[CIT0068] Wurm, S., Diehl, M., Kornadt, A. E., Westerhof, G. J., & Wahl, H. W. (2017). How do views on aging affect health outcomes in adulthood and late life? Explanations for an established connection. Developmental Review, 46, 27–43. doi:10.1016/j.dr.2017.08.002 33927468 PMC8081396

[CIT0069] Wurm, S., Wiest, M., Wolff, J. K., Beyer, A. K., & Spuling, S. M. (2020). Changes in views on aging in later adulthood: The role of cardiovascular events. European Journal of Ageing, 17(4), 457–467. doi:10.1007/s10433-019-00547-5 33380999 PMC7752931

