# Supplementary material for: Feeling Younger in Rural Burkina Faso: Exploring the Role of Subjective Age in the Light of Previous Research From High-Income Countries
Source: J Gerontol B Psychol Sci Soc Sci. 2021 Aug 11;76(10):2029–40. doi: 10.1093/geronb/gbab151 (PMC8599075; doi:10.1093/geronb/gbab151)
Supplement: Supplementary file 1 [file gbab151_suppl_supplementary_materials_1.pdf]

Supplementary Material

Supplementary Table 1

Information on Missing Values by Variable (N=3028).

Variable	n (%) missing
Chronological age	2 (<1%)
Subjective age (prop. score)	74 (2%)
Felt age	74 (2%)
Household size	0 (0%)
Education level	0 (0%)
Walk speed	98 (3%)
PHQ-9 score (depression)	0 (0%)
CSID score (cognition)	0 (0%)
WHOQOL score	0 (0%)

Note: Subj. Age (prop.)= (Felt age – chronological age) / (chronological age); Felt age= How old do you feel (years)?; PHQ-9= Patient health questionnaire (9-item version); CSI-D= Community screening instrument for dementia; WHOQOL= WHO quality of life scale (normalized).

Supplementary Table 2

Zero-Order Correlations of the Study's Major Variables (N= 3028).

Variables	1.	2.	3.	4.	5.	6.	7.	8.	9.	10.
1. Subjective age (prop. score)	1									
2. Felt age	.56***	1								
3. Chronological age	.05**	.85***	1							
4. Sex	.08***	.13***	.10***	1						
5. Household size	-.02	-.08***	-.09***	-.05**	1					
6. Education level	-.03	-.13***	-.14***	-.16***	-.09***	1				
7. Walk speed	-.13***	-.35***	-.34***	-.31***	.05**	.15***	1			
8. PHQ-9 score (depression)	.19***	.36***	.32***	.16***	-.06***	-.09***	-.21***	1		
9. CSID score (cognition)	-.12***	-.29***	-.27***	-.15***	.08***	.12***	.19***	-.18***	1	
10. WHOQOL score	-.16***	-.37***	-.34***	-.16***	.13***	.10***	.24***	-.42***	.24***	1

Note: Subj. Age (prop.)= (Felt age – chronological age) / (chronological age); Felt age= How old do you feel (years)?; PHQ-9= Patient health questionnaire (9-item version); CSI-D= Community screening instrument for dementia; WHOQOL= WHO quality of life scale (normalized).

Missing data handled by pairwise deletion.

*p < .05. **p < .01. ***p < .001

Supplementary Figure 1

Discrepancy between Chronological Age and Felt Age across the Span of Chronological Age in the Nouna Study (N = 3028) Separate for A) Male and B) Female Participants.

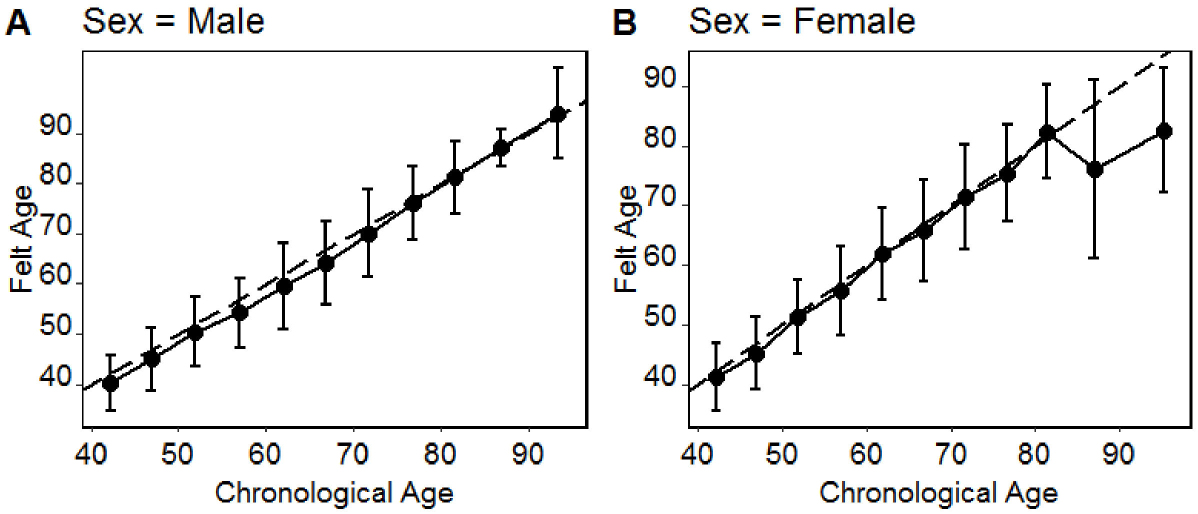

Note: Dashed line functions as reference that indicates Felt Age (in years; y-axis) equal to Chronological Age (in years; x-axis). In both A) and B) dots are means and whiskers show the standard deviation. Before calculation of these descriptive summary measures participants were grouped by chronological age (in bins of 5 years).

Supplementary Figure 2

Associations Between Major Study Variables Estimated Using Linear and Non-Linear Regression Models.

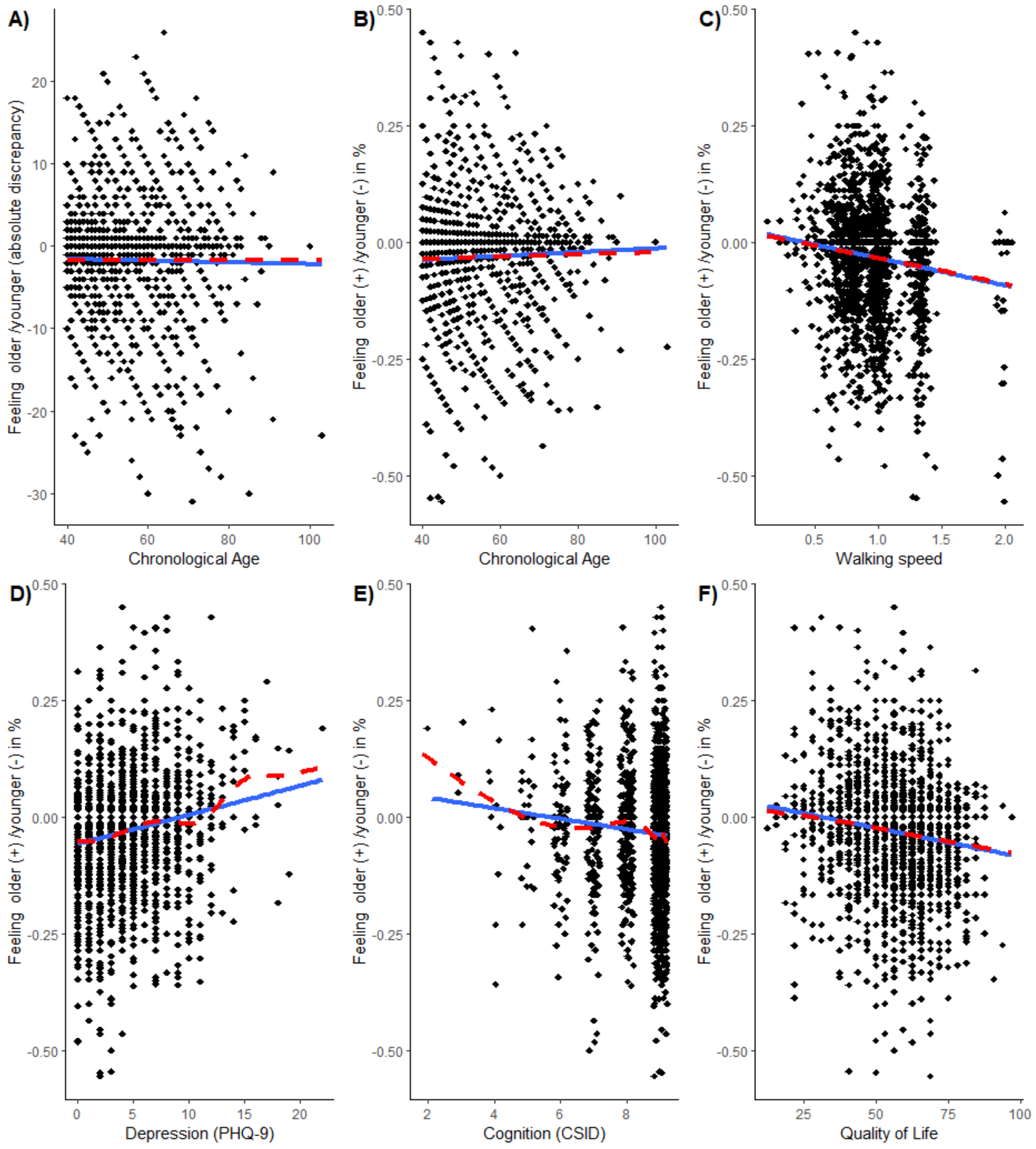

Note: In all panels the blue line shows the simple linear regression, whereas the red dashed line is based on a non-linear (LOESS) method. Missing data was handled by pairwise deletion.

Supplementary Figure 3

Subjective Age (Proportional Score) and Subjective Health.

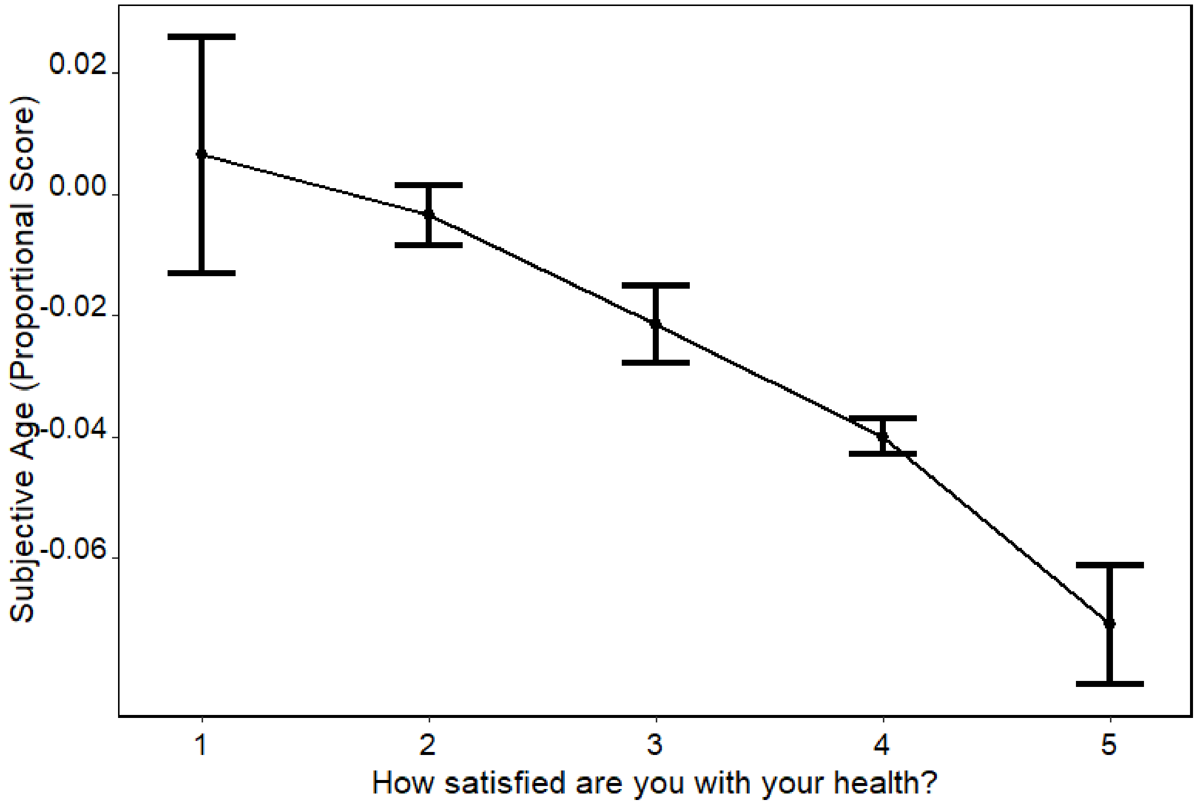

Note: Subjective age as proportional score (y-axis) and response to item #2 of the WHOQOL indicating subjective health (x-axis, "How satisfied are you with your health?"). Subjective health was rated on a 5-level Likert scale with higher values indicating stronger satisfaction with health.

French Version of Subjective Age 1-Item Assessment and AARC 10-Item Questionnaire

Vieillissement subjectif

1. Beaucoup de gens se sentent plus vieux ou plus jeunes qu'ils ne le sont en réalité. En général, vous vous sentez comme quelqu'un de combien des ans ?
    a. ______ ans
